# Current News

**Published:** 1899-11

**Authors:** 


### Current News.

### PENNSYLVANIA BOARD OF DENTAL EXAMINERS.

The Pennsylvania Board of Dental Examiners will conduct examinations simultaneously in Philadelphia and Pittsburg, December 18, 19, and 20, 1899.

Application for examination must be made to Hon. James W. Latta, Secretary of the Dental Council, Harrisburg, Pa.

G. W. Klump,
*Secretary.*

Williamsport, Pa.

